# The Average Mutual Information Profile as a Genomic Signature

**DOI:** 10.1186/1471-2105-9-48

**Published:** 2008-01-25

**Authors:** Mark Bauer, Sheldon M Schuster, Khalid Sayood

**Affiliations:** 1 Department of Electrical Engineering, University of Nebraska, Lincoln, NE USA; 2 Keck Graduate Institute, Claremont Colleges, Claremont, CA, USA

## Abstract

**Background:**

Occult organizational structures in DNA sequences may hold the key to understanding functional and evolutionary aspects of the DNA molecule. Such structures can also provide the means for identifying and discriminating organisms using genomic data. Species specific genomic signatures are useful in a variety of contexts such as evolutionary analysis, assembly and classification of genomic sequences from large uncultivated microbial communities and a rapid identification system in health hazard situations.

**Results:**

We have analyzed genomic sequences of eukaryotic and prokaryotic chromosomes as well as various subtypes of viruses using an information theoretic framework. We confirm the existence of a species specific average mutual information (AMI) profile. We use these profiles to define a very simple, computationally efficient, alignment free, distance measure that reflects the evolutionary relationships between genomic sequences. We use this distance measure to classify chromosomes according to species of origin, to separate and cluster subtypes of the HIV-1 virus, and classify DNA fragments to species of origin.

**Conclusion:**

AMI profiles of DNA sequences prove to be species specific and easy to compute. The structure of AMI profiles are conserved, even in short subsequences of a species' genome, rendering a pervasive signature. This signature can be used to classify relatively short DNA fragments to species of origin.

## Background

The existence of patterns that can be used as a signature of data is indicative of statistical or deterministic structures in the data. In DNA sequences this structure can be due to biological processes which involve the DNA or they may appear because of events and processes in the evolutionary history of the DNA. There have been significant efforts in understanding the sequential structure and complexity of DNA using various approaches, information theoretic measures or other mathematical models.

The standard approach to studying statistical relationships in a sequence is the use of correlation profiles or spectral profiles such as periodograms and power spectrums. To translate the sequence of letters that form the DNA sequence into a sequence of numbers, which can then be easily analyzed using autocorrelation or spectral techniques, different mappings have been proposed by Gates [1], Voss [2] and Peng et al. [3]. The power spectral densities obtained from these approaches show a power law relationship, which points to the existence of long range correlations. A number of models have been proposed to account for these long range correlations [4,5].

Somewhat distinct from statistical models, several researchers have used information theoretic measures to study the characteristics of the DNA sequence. A description of the use of information measures to study DNA sequences can be found in Gatlin [6] and Roman-Roldan et al. [7]. Schneider et al. [8-10] have used information theoretic measures in a number of interesting ways from studying the information content at nucleotide binding sites to expediting alignment. However, most of the applications of information theory has been to the study of the correlation properties of DNA sequences [7,11-14]. A significant portion of these are directed toward obtaining a mechanism for the long range correlation properties of the DNA sequence, while others study the ability of information theoretic measures to differentiate between coding and non-coding regions or to demonstrate a close relationship between sequence compositional complexity of the DNA sequence and the biological complexity of the organism to which the sequence belongs [15,16].

Another line of approach to understand the compositional structure of DNA sequences has focused on frequency profiles of short oligonucleotides. Karlin and co-workers [17-19] have shown that there is a compositional bias in bacterial genomic sequences. Blaisdell and co-workers have shown the same for viral sequences [20]. Karlin et al. [21] have used this compositional bias in bacterial genomes to infer evolutionary relationships. The compositional biases of DNA sequences have also been studied from the point of view of linguistics. Brendel et al. [22,23] provide a technique to identify possible short oligonucleotide sequences within DNA sequences based on the deviation of the frequency of occurrence of these sequences from their expected value. Bultrini et al. [24] propose the existence of a pentamer vocabulary characterizing intron and intron-like intergenic tracts. This approach has been used for intron/exon discrimination as well as for gene finding.

One important implication of different approaches to characterize the structure and complexity of DNA sequences has been the interest in discovering patterns in genomic sequences that can be used as signatures of species. Such signatures can be useful in a wide variety of contexts. If differences between signatures can be related to evolutionary distance they can be used for developing phylogenetic relationships and for understanding evolutionary processes [18,20,21,25].

The existence of reliable genomic species signatures would have significant implications in developing a rapid identification system using DNA sequences. Bacterially transmitted diseases continue to be a major threat to health with increasing threat from previously unknown variants, which have antibiotic resistance. The threat of bioterrorism adds to this potentially lethal mix. In order to respond to a disease outbreak, whether initiated by natural or artificial means, there is an urgent need for rapid identification of infectious agents to limit exposure and initiate treatment. Therefore, it is important to identify and understand structures within the genome of organisms which differentiate them from each other and from more benign organisms.

The recent presentation of the genomic sequences of large microbial populations presents yet another application for a species signature [26,27]. Tyson et al. used random shotgun sequencing of DNA from a natural acidophilic biofilm to identify the structure of the uncultivated microbial community [26]. In a similar approach Venter et al. targeted a much more complex microbial population collected from the Sargasso Sea region [27]. In this latter study, approximately 3 million reads yielding about 1.6 billion base pairs of DNA sequences were generated. It is believed that these sequences belong to at least 1,800 genomic species. These approaches present a very complicated problem of identification and assembling shotgun reads coming from an unknown number of species. Signatures which can be used to identify and distinguish between fragments based on their species of origin would be useful in this process.

Most existing approaches to defining species specific signatures are based on frequency distribution of oligonucleotides, also referred to as "words" [28-30]. However, the choice of the length of the words and the DNA sequence window in which the frequency profiles of the words are observed not only result in data explosion but also change the composition of the resulting signature. In this paper we present AMI profile of DNA sequences as a candidate for species signature. AMI profiles are pervasive in the sense that they can be detected in small fragments of the DNA sequence. The proposed genomic signature is a vector where the *k*^*th *^entry is the AMI between nucleotides that are *k *locations apart. AMI profiles are generated virtually free form any parameters resulting in an automated unbiased calculation. We also use this signature to develop a simple, computationally inexpensive measure of distance between genomic sequences. We validate this distance measure by using it with standard phylogenetic algorithms to perform unsupervised clustering.

AMI was first introduced for studying the communication of signals under noisy channel conditions [31]. In communication theory it is interpreted as a measure of the information contained in one event *X *about another event *Y *(or vice versa). In the bioinformatics area the average mutual information has been used to detect correlated mutations at noncontiguous sites in a sequence [13], for secondary structure prediction [32,33] to investigate correlations between sites in protein sequences [7,11,12], and to differentiate between coding and noncoding regions [34]. Slonim et al. [35] use average mutual information to formulate the clustering problem in a variety of settings including gene expression, stock prices, and movie ratings. Slonim et al. [36] also use average mutual information to study the relationships between genes and their phenotypes.

Berryman et al. [37] have used the average mutual information profile to demonstrate long-range correlation in DNA sequences. More important, from the perspective of this work, they show that the long-range structures evident in the profile of a sequence results from evolutionary events such as additions, deletions, and insertions of repetitive elements. This view is further validated by the work of Holste et al. [38] which focuses on two specific peaks at *k *= 135 and *k *= 160 in the average mutual information profile of Human Chromosomes 20, 21, and 22. When they replace *Alu *repeats in the chromosomes with random sequences these peaks disappear validating their contention that the peaks occur due to the presence of *Alu *repeats. The discrimination property of AMI was also demonstrated by Dehnert et al. [39,40] for eukaryotic chromosomes. Dehnert et al. [39] use the Euclidean distance between AMI profiles and coefficients of autoregressive models to discriminate between various eukaryotic genomes. Hummel et al. [41] use average mutual information to analyze protein sequence motifs. In the work of Hmmel et al., as in earlier works [13,42] the different sequences are first aligned using a multiple sequence alignment and treated as realizations of a random process. The probabilities needed to compute the average mutual information are then obtained from this ensemble.

These results indicate that on some level the AMI profile can be viewed as a representation of the evolutionary history of the organism. As the AMI profile is an *average *measure the structure evinced by the profile is likely to be pervasive. That is, this history should be reflected to some extent in all parts of the genome and sufficiently long fragments of the genome should have similar profiles. Organisms that are evolutionarily related have an extensive common history. If the AMI profile reflects evolutionary history, this common history should be reflected in similarity of their AMI profiles. In the following we present evidence to support this hypothesis, based upon which we suggest that the AMI profile is an excellent candidate for a species signature.

## Results

### The AMI profile of chromosomes

**Figure 1:**
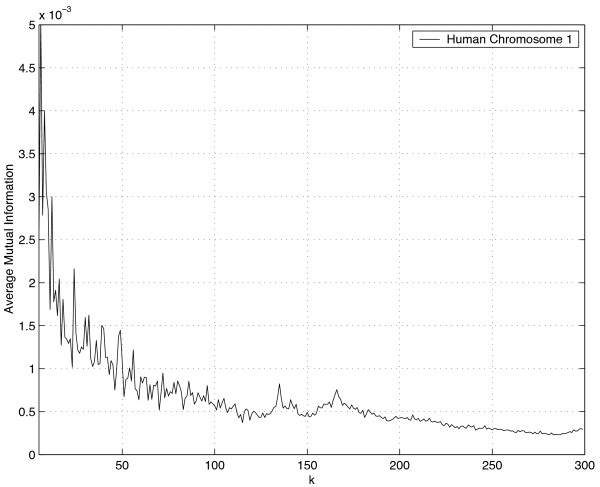
Average Mutual Information Profile for Human Chromosome 1 plotted for *k *≥ 5. The x-axis is the distance between bases while the y-axis is the value of the average mutual information *I*_*k*_.

We begin with the largest fragments of available DNA sequences, the chromosomes of eukaryotes. Consider the AMI profile shown in Figure 1 corresponding to Human chromosome 1. The abscissa corresponds to the distance between two bases in the sequence, while the ordinate is the value of the average mutual information. A larger value of the average mutual information for a particular value of *k *corresponds to higher dependence between bases *k *apart. Clearly we would expect higher dependence between bases closer than between bases further apart. The various peaks may be the result of a number of factors including the ratio of coding to noncoding regions and the existence of various kinds of repeats.

**Figure 2:**
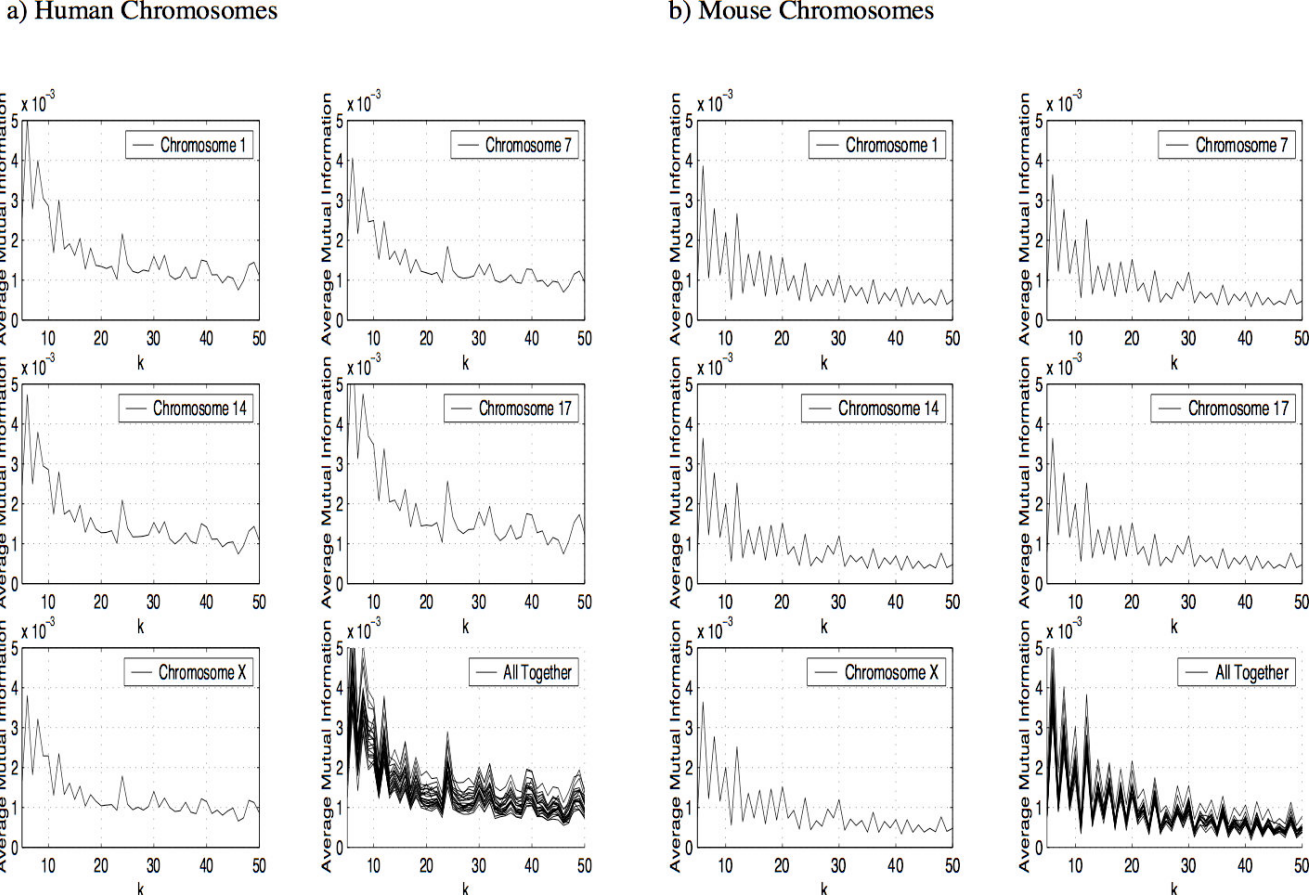
Average Mutual Information Profile for the Human Chromosomes plotted for values of *k *between 5 and 50, b) Average Mutual Information Profile for the Mouse Chromosomes plotted for values of *k *between 5 and 50.

If we now plot the AMI profile for different chromosomes as shown in Figure 2a, we see that the peaks and valleys occur at identical locations. This is true of all chromosomes in spite of the significant differences in size and gene content. We have plotted the AMI profile for values of *k *between 5 and 50 to better show the similarities. The same holds true for other values of *k*. Note that we have not tried to align the chromosomes which, given their diversity, would not have been feasible. Plotting the same chromosomes for mouse (*mus musculus*) in Figure 2b, we see again the similarity between the AMI profiles for the various chromosomes. We can also see that these profiles are distinct from those of the human chromosomes.

**Figure 3:**
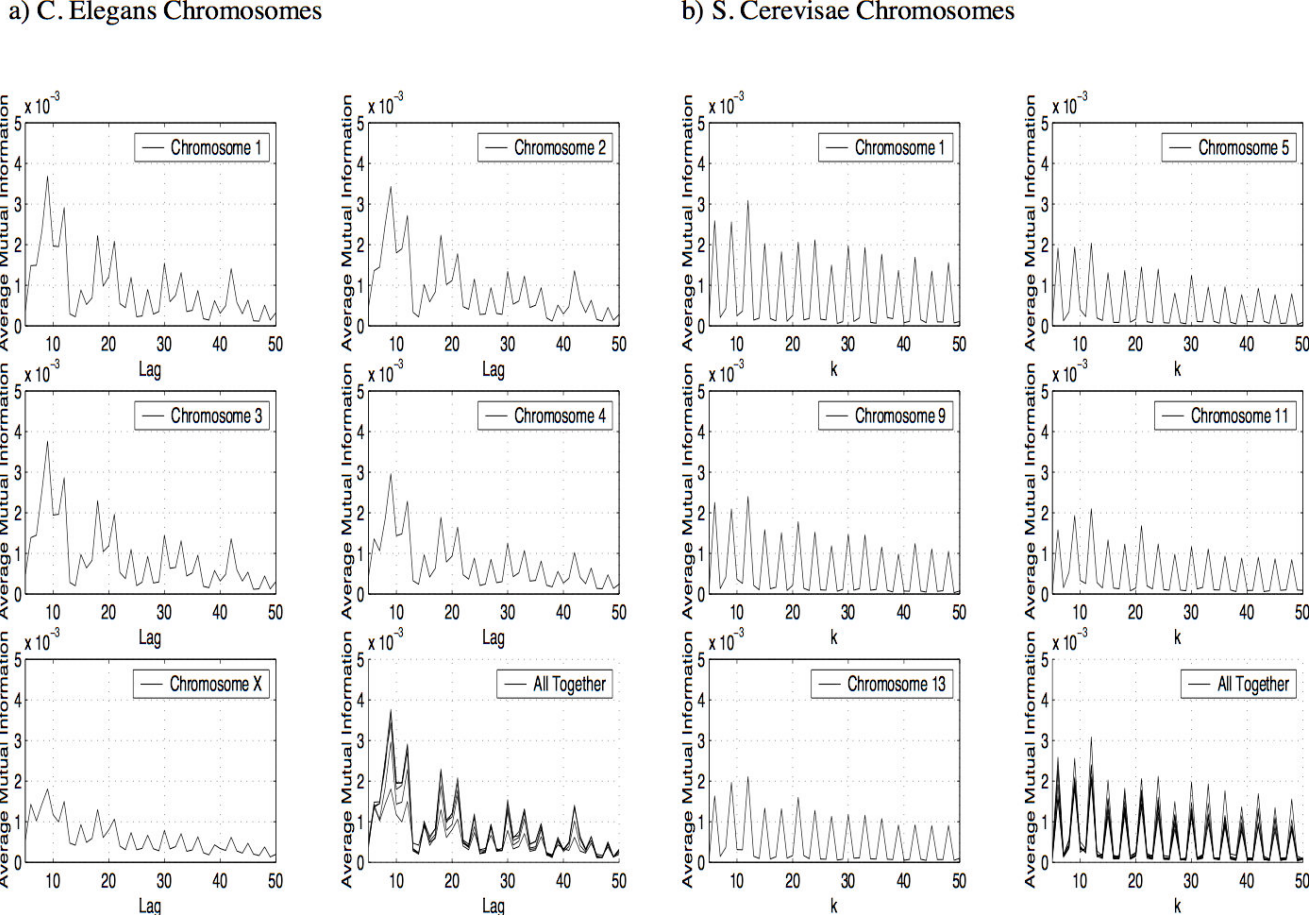
Average Mutual Information Profile for the C. elegans Chromosomes plotted for values of *k *between 5 and 50, b) Average Mutual Information Profile for the S. cerevisiae Chromosomes plotted for values of *k *between 5 and 50.

If we repeat this experiment for the chromosomes of *C. elegans *we get the same result. Again, when we plot the profile we see a pattern of peaks and valleys which occur at the identical locations for all chromosomes of *C. elegans*. We demonstrate this with five chromosomes of *C. elegans *in Figure 3a. Again, while the pattern of peaks and valleys in the AMI profile is the same for all chromosomes of *C. elegans*, this pattern is distinctly different from the pattern of peaks and valleys in the human and mouse AMI profiles.

Finally we repeat the experiment for *Saccharomyces cerevisiae*. The results are shown in Figure 3b (note the peaks at multiples of three reflecting a larger proportion of coding regions compared to the previous examples). Once more we obtain a sequence of peaks and valleys in the AMI profile which are the same for all chromosomes of *S. cerevisiae*, and this pattern of peaks and valleys is different from the patterns in the profiles of the other species.

**Figure 4:**
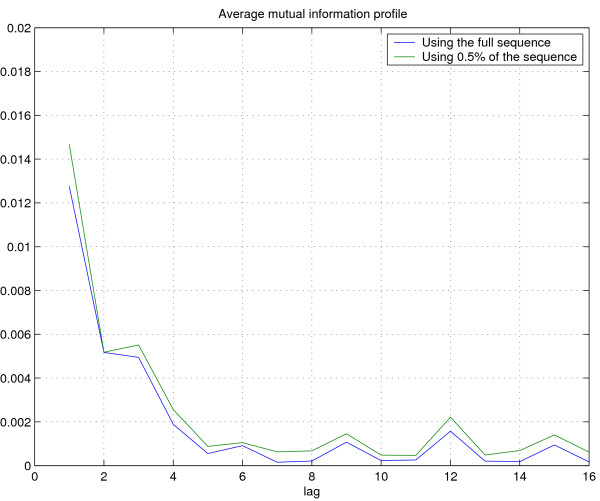
Plot of the first sixteen elements of the average mutual information profile for *E. coli *using the entire sequence and using 0.5% of the sequence.

We then plot AMI profiles for the complete *E. coli *sequence (accession number NC_000913) and a 0.5% fragment of the sequence in Figure 4 to check for pervasiveness. The striking similarity between the profile suggests that AMI profiles can be used to identify random fragments of a DNA sequence with their species of origin.

**Figure 5:**
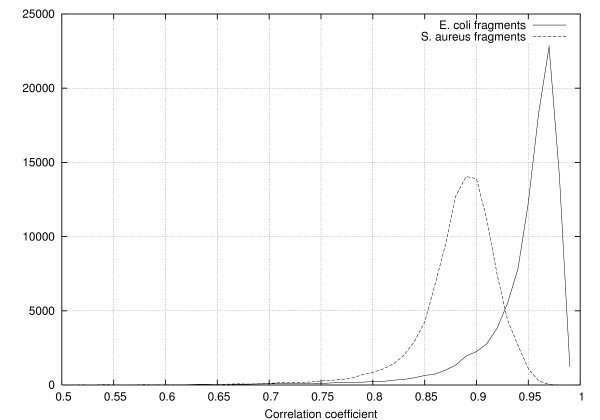
Plot of the histogram of the correlation between the average mutual information profile of fragments of *E. coli *and *S. aureus *with the average mutual information profile of the entire *E. coli *genome.

We test this hypothesis by computing the correlation coefficient of the AMI profile of 100,000 5 kb long fragments of the *E. coli *genome with the AMI profile of the entire sequence. We also compute the correlation coefficient of 100,000 random fragments from the *S. aureus *genome (accession number NC_002758) with the AMI profile of the *E. coli *genome. The histograms of the correlation coefficient are shown in Figure 5. The results clearly demonstrate both the pervasiveness of the AMI signature as well as its specificity.

**Figure 6:**
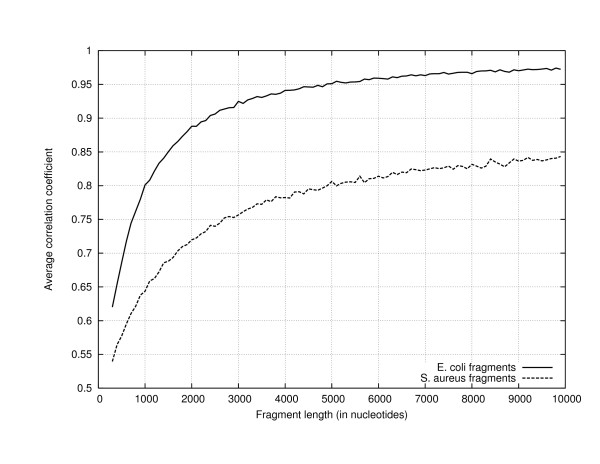
Plot of the average correlation between the average mutual information profile of fragments of *E. coli *and *S. aureus *with the average mutual information profile of the entire *E. coli *genome as a function of fragment length. The average correlation was obtained using 1000 trials with the appropriate fragment length.

Finally, to investigate the length of fragment required to compute a genomic signature we plot the average correlation of profiles of 1000 fragments of genomic DNA with a reference profile obtained from the entire genome in Figure 6. The size of the fragments are varied from 200 nucleotides to 10,000 nucleotides. In these experiments we have restricted the size of the AMI profile to sixteen in order to easily compute the profiles of short segments. The reference AMI profile is that of *E. coli *and the fragments are from *E. coli *and *S. aureus*. As was to be expected, the plot shows that the correlation between the profiles of the fragments and the reference profile of the genome increases with increasing fragment length. While this is true for profiles of both *E. coli *and *S. aureus *fragments, the profiles of the *E. coli *fragments are consistently more correlated with the reference profile than the profiles of the S. aureus fragments. This is true for all fragment sizes. This suggests that the AMI profile could be useful in classifying relatively short fragments. All these figures indicate the existence of a profile specific to a species. Using this as our motivation we develop a distance measure which can be used to classify genomic sequences to species of origin. We verify the utility of this metric by classifying retroviruses based on their host species and by classifying subtypes of the HIV-1 virus.

### A distance measure

Noting that genomic sequences from the same species have similar pattern of peaks and valleys a numerical measure of the closeness of their AMI profiles can be obtained by looking at the correlation coefficient between the AMI profiles. As the larger values of the AMI profile for small values of *k *tend to mask the differences between AMI profiles we evaluate the correlation coefficient for values of *k *greater than 5. In our simulations the upper limit for *k *was 512. Using values of *k *greater than 512 did not effect the results. We define the distance *d*_*ij *_between AMI profiles of the *i*^*th *^and *j*^*th *^sequences to be one minus the correlation coefficient. Note that to compute distances between sets of sequences we do not need to align these sequences. This is especially useful when we look at distances between chromosomes as multiple sequence alignment for whole genomes or chromosomes is an unsolved problem. The availability of an alignment-free approach to finding the distance between genomic sequences may considerably simplify the investigation of genomic relatedness of species based on their sequence information.

We apply this distance measure to three chromosomes from four species. The particular chromosomes are listed in Table 1. The distances between these chromosomes are shown in Table 2. Clearly, the distances between chromosomes from the same species are substantially smaller than the distances between chromosomes of different species. Furthermore the distances between the AMI profile of chromosomes of more closely related species such as mouse and human is substantially less than the distance between less closely related species such as mouse and yeast. For the species for which we have sequences available the pattern holds for other chromosomes as well.

**Table 1:** Labels for chromosomes

	Accession	Chromosome
*m*14	NT 002582	M. musculus chromosome 14
*m*17	NT 002588	M. musculus chromosome 17
*MX*	NT 003030	M. musculus chromosome X
*sc*3	NC 001135	S. cerevisiae chromosome 3
*sc*5	NC 001137	S. cerevisiae chromosome 5
*sc*9	NC 001141	S. cerevisiae chromosome 9
*ce*1	NC 000965	C. elegans chromosome 1
*ce*2	NC 000966	C. elegans chromosome 2
*ce*3	NC 000967	C. elegans chromosome 3
*h*14	NT 003140	H. sapiens Chromosome 14
*h*17	NT 002831	H. sapiens Chromosome 17
*HX*	NT 001374	H. sapiens Chromosome X

Labels used for the chromosomes of various species in Table 2.

**Table 2:** Distance between chromosomes.

	*sc*9	*sc*5	*sc*3	*mX*	*m*17	*m*14	*hX*	*h*17	*h*14	*ce*3	*ce*2	*ce*1
*sc*9	0.000	0.018	0.017	0.512	0.485	0.513	0.549	0.539	0.536	0.377	0.373	0.355
*sc*5	0.018	0.000	0.016	0.446	0.418	0.450	0.483	0.469	0.469	0.312	0.309	0.291
*sc*3	0.017	0.016	0.000	0.459	0.433	0.461	0.496	0.485	0.483	0.339	0.334	0.317
*mX*	0.512	0.446	0.459	0.000	0.009	0.015	0.046	0.055	0.056	0.205	0.197	0.205
*m*17	0.485	0.418	0.433	0.009	0.000	0.029	0.063	0.066	0.074	0.209	0.202	0.208
*m*14	0.514	0.450	0.461	0.015	0.029	0.000	0.071	0.083	0.079	0.225	0.216	0.225
*hX*	0.549	0.483	0.496	0.046	0.063	0.071	0.000	0.003	0.002	0.197	0.186	0.199
*h*17	0.539	0.469	0.485	0.055	0.066	0.083	0.003	0.000	0.004	0.189	0.179	0.189
*h*14	0.536	0.469	0.483	0.056	0.074	0.079	0.002	0.004	0.000	0.188	0.178	0.189
*ce*3	0.377	0.312	0.339	0.205	0.209	0.225	0.197	0.189	0.188	0.0000	0.002	0.003
*ce*2	0.373	0.309	0.334	0.197	0.202	0.216	0.186	0.179	0.178	0.002	0.000	0.004
*ce*1	0.355	0.291	0.317	0.205	0.208	0.225	0.199	0.189	0.189	0.003	0.004	0.000

Distance between the profiles of *Mus musculus, Saccharomyces cerevisiae, C. elegans*, and *Human *chromosomes

**Figure 7:**
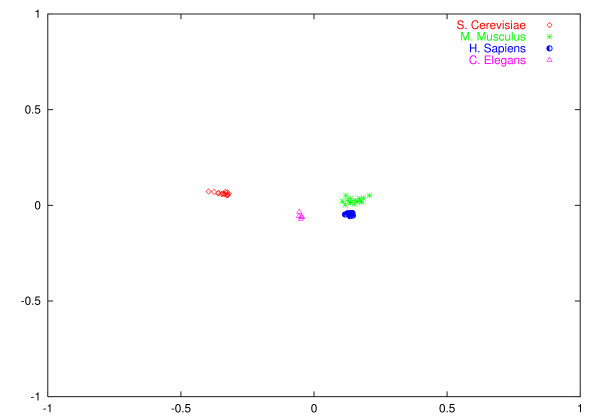
Clustering of all chromosomes from *S. cerevisiae*, *M. musculus, H. sapiens *and *C. elegans*. The clustering and visualization approach is described in the Methods section.

It is difficult to show the data for all the chromosomes in tabular form. We have developed a visualization program (described in Methods), which gives a visual representation of the distances between AMI profiles. One representation of the distances of the chromosomes of the four species used in this experiment is shown in Figure 7. Note that as we are projecting from a multi-dimensional space into a two-dimensional space the representation is not unique. However, in order to show that a population can be separated into different classes all we need to show is clustering in a single representation. That all the genomic sequences can be assigned to their particular species is clear from the figure. The program provides a means of visualizing the distances between AMI profiles and qualitative evidence for clustering. We can also show visual evidence of clustering using a singular value decomposition. In the next section we show that these distances can be used in a quantitative manner with the UPGMA algorithm to provide unsupervised clustering.

### Grouping HIV subtypes

**Figure 8:**
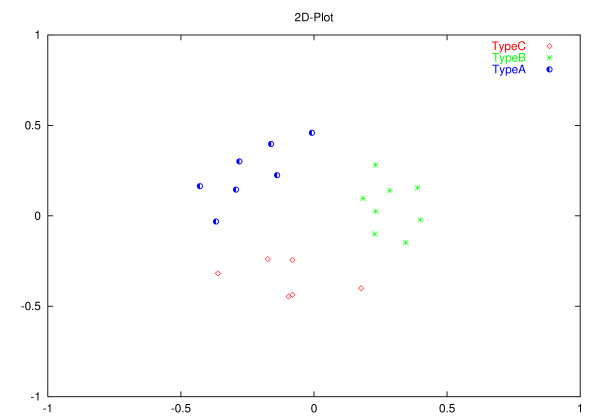
Clustering of HIV-1 subtypes based on the distance between their respective AMI profiles.

**Figure 9:**
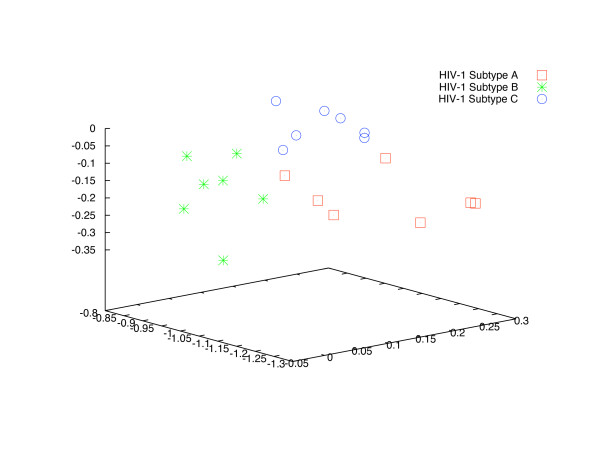
Clustering of HIV-1 subtypes evidenced by three coefficients of the singular valued decomposition of the AMI profiles.

**Figure 10:**
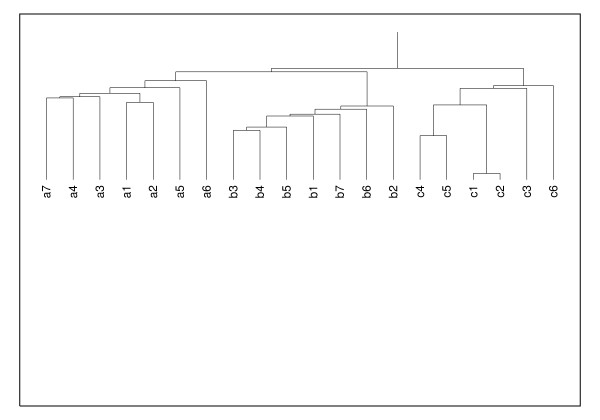
UPGMA tree for subtypes of the HIV-1 virus. The distances used to construct the UPGMA tree were obtained from their respective AMI profiles. The labels used here are defined in Table 3.

The Human immunodeficiency viruses (HIV) represent a group of retroviruses that are distinct from endogenous retroviruses and are not presumed to have originated from human cellular DNA sequences. However, the life cycle of these viruses and their genome are essentially the same as that of all other retroviruses – reverse transcription of the RNA genome into proviral DNA followed by integration into host cell chromosomal DNA and the formation of progeny viral RNA genome by transcription from the proviral DNA. Of the two major types of the HIV virus, HIV-1 is the more virulent and is the predominant strain. There are multiple subtypes of the HIV-1 virus with some degree of geographic segregation between the various subtypes. This geographic segregation argues for evolutionary differences between the different subtypes. As such, it should be feasible to differentiate between the different subtypes using the AMI profile. The results of our analysis of AMI profiles of the genomes of twenty one independent viral isolates listed in Table 3 are shown in Figure 8. The clustering approach used is described in the Methods section. We also show clustering by plotting three coefficients from the singular value decomposition of the AMI profiles in Figure 9. The UPGMA tree, constructed using the distance measure described earlier, corresponding to these isolates is shown in Figure 10.

**Table 3:** Labels for HIV subtypes

	Acc. No.	Description
*a*1	AF004885	HIV-1 isolate from Kenya (Subtype A)
*a*2	AF069671	HIV-1 isolate from Sweden, (Subtype A)
*a*3	U51190	HIV-1, isolate from Uganda (Subtype A)
*a*4	AF069672	HIV-1 isolate from Sweden (Subtype A)
*a*5	AF107771	HIV-1 isolate from Sweden (Subtype A)
*a*6	M62320	HIV-1 Ugandan isolate (Subtype A)
*a*7	AF069670	HIV-1 isolate from Somalia (Subtype A)
*b*1	AF042101	HIV-1 isolate from Australia (Subtype B)
*b*2	U37270	HIV-1 isolate from Australia (Subtype B)
*b*3	U43096	HIV-1 isolate from Germany (Subtype B)
*b*4	U43141	HIV-1 isolate from Germany (Subtype B)
*b*5	AJ006287	HIV-1 isolate from Spain (Subtype B)
*b*6	AF146728	HIV-1 from Australia (Subtype B)
*b*7	U71182	HIV-1 isolate from China (Subtype B)
*c*1	AF110960	HIV-1 isolate from Botswana (Subtype C)
*c*2	AF110959	HIV-1 isolate from Botswana (Subtype C)
*c*3	U52953	HIV-1 isolate from Brazil (Subtype C)
*c*4	AF067157	HIV-1 isolate from India (Subtype C)
*c*5	AF067155	HIV-1 isolate 21068 from India (Subtype C)
*c*6	U46016	HIV-1 Human immunodeficiency virus type 1 (subtype C)
*c*7	AB023804	HIV-1 Human immunodeficiency virus type 1 (subtype C)

List of accession numbers, descriptions, and labels of HIV 1 sequences used to examine distances between subtypes.

The distance between members of each subgroup is relatively high as compared to DNAs of different chromosomes of the same species. However, the distance between AMI profiles from different subgroups is higher than between members of the same subgroup. This is clear from the clustering evident in Figure 8, Figure 9 and from the UPGMA tree shown in Figure 10. The AMI profile and the proposed distance measure may, therefore, allow functional distinction between genomes that are evolutionarily comparable but have acquired new biological characteristics.

## Discussion

The observations reported here suggest that the average mutual information analysis of long range genomic structure can yield new insight into the nature of the genome. The data reported here indicate that entire genomic sequences can be analyzed (without the need for multiple alignments) in efforts to gain an understanding of the evolutionary relationship between various species, and among chromosomes within a single species.

As described here, the average mutual information profile of genomic structure reveals a great deal of fine structure in the various sequences available. This structure might possibly have been ignored, except for the fact that so much is highly reproducible among the various chromosomes. Based on the fact that the distances between AMI profiles seem to correlate with evolutionary relationship we speculate that the structure revealed by the average mutual information profiles is closely related to the evolution of various species and their genomes. Finally, as the AMI profile for each sequence is obtained without reference to other sequences there is no need for a multiple sequence alignment when comparing sequences.

## Conclusion

The AMI profile provides a simple, easily computable, species signature. The signature can be used in applications where evolutionary relationships need to be deduced using relatively short fragments of DNA as well as where evolutionary relationships between organisms are to be studied using large genomic sequence. Distances between sets of genomic sequences can be obtained without the need for multiple sequence alignment. The profile, and the distance measure associated with it, may also be useful, either by itself or in conjunction with other signatures, to discriminate between fragments of DNA from different species and to identify fragments of genomic DNA with the species of origin.

## Methods

### AMI profile for DNA sequences

In this work we examine a particular information theoretic measure, average mutual information, as a candidate for species signature. If we have two events *X *and *Y *which are independent of each other then the joint probability of occurrence of the two events, *p*(*X*, *Y*) is simply the product of the probability of occurrence of each event, *p*(*X*, *Y*) = *p*(*X*)*p*(*Y*). Thus, the deviation from unity of the ratio *p*(*X*, *Y*)/[*p*(*X*)*p*(*Y*)], or the deviation from zero of the logarithm of this ratio, can be used as a measure of dependence. If we take *X *to be the base at some location and *Y *to be the base at location *k *downstream from it we can define an average measure of dependence as:

$$I_{k}=\sum_{X\in A}\sum_{Y\in A}p_{k}(X,Y)\log\frac{p_{k}(X,Y)}{p(X)p(Y)}$$

where A is the set of nucleotides {*A*, *G*, *C*, *T*}. We have added the subscript *k *to the joint probability to show that the nucleotides occur *k *bases apart. By plotting the average mutual information for different values of *k *we can arrive at a profile for a particular sequence. We refer to this profile as the *average mutual information (AMI) profile*.

We compute the average mutual information for bases *k *apart by estimating the probabilities using the relative frequencies of occurrence. Let *n*_*k*_(*X*, *Y*) be the number of times two bases *k *apart take on the values *X *and *Y*, where *X *and *Y *can be *A*, *C*, *G*, and *T *. The joint probabilities *p*_*k*_(*X*, *Y*) are estimated by

$$p_{k}(X,Y)=\frac{n_{k}(X,Y)}{\sum_{I\in A}\sum_{J\in A}n_{k}(I,J)}$$

The marginal probabilities *p*(*X*) can similarly be estimated by dividing the total number of times the nucleotide *X *occurs divided by the total number of bases in the sequence.

### The visualization program

The visualization program was developed in order to visualize the distances between a large number of multidimensional vectors. The particular application was to visualize the distance between AMI profiles of a large number of DNA sequences.

The program requires as its input a list of the sequences {*s*_*i*_} and their distances {*d*_*i*,*j*_} from each other. The user can also input just the AMI profile of the sequences. The program then calculates the distances. These distances are defined earlier in the paper. Each sequence is treated as a point in a two or three dimensional free space which is operated on by "forces" exerted upon it by the points representing all other sequences.

The points corresponding to the sequences are initially assigned a random locations *l*_*i *_in the unit square or cube and a random velocity *v*_*i*_. For each sequence *s*_*i *_a vector "force" *f*_*i*,*j *_due to all other sequences *s*_*j *_is calculated. The force is defined as

$$f_{i,j}=(d_{i,j}-{\hat{d}}_{i,j})u_{j,i}$$

where ${\hat{d}}_{i,j}$ is the Euclidean distance between the assigned locations of *s*_*i *_and *s*_*j *_and **u**_*j*,*i *_is the unit vector from *s*_*j *_to *s*_*i*_. The cumulative force on *s*_*i *_is calculated as

$$f_{i}=\sum_{j}f_{i,j}$$

This force is calculated for each of the sequences. The vector velocities of the sequences are then updated by displacing them by an amount proportional to the vector force on them. The update equation for sequence *s*_*i *_at time *n *+ 1 is given by

$$v_{i}^{\left(n+1\right)}=\alpha (v_{i}^{\left(n\right)}+\beta f_{i}^{\left(n\right)})$$

where the superscripts denote the iteration counter. The locations of the sequences are then updated by displacing them by an amount proportional to the vector velocities.

$$l_{i}^{\left(n+1\right)}=l_{i}^{\left(n\right)}+\gamma v_{i}^{\left(n+1\right)}$$

The constants *α*, *β*, and *γ *were experimentally determined to provide a good tradeoff between rate of convergence and jitter. A larger value for these constant will permit a faster convergence with considerable jitter around the final configuration, and vice versa. We picked the constants to be 0.7, 0.1, and 0.1. After the locations are updated the process is repeated until the sequences have stabilized in their locations. Our observation is that it requires about fifty iterations for the configuration to stabilize. Keeping in mind that there are multiple stable configurations and to prevent the system from settling into a local minimum, we randomly perturb the configuration every 50 updates. The size of the random perturbations is uniformly distributed in the interval [-.5,.5] and is multiplied by 0.95^*m *^where *m *is the number of random perturbations applied to this point. The 2D version of the program is available for use at [43].

## Authors' contributions

KS conceived the study, and together with MB designed and tested the algorithm. SS provided the biological insight. All authors read and approved the final manuscript.
